# Costs of weaponry: Unarmed males sire more offspring than armed males in a male‐dimorphic mite

**DOI:** 10.1111/jeb.13402

**Published:** 2018-12-03

**Authors:** Tom P. G. Van den Beuken, Chris C. Duinmeijer, Isabel M. Smallegange

**Affiliations:** ^1^ Department of Evolutionary and Population Biology Institute for Biodiversity and Ecosystem Dynamics University of Amsterdam Amsterdam The Netherlands

**Keywords:** Acaridae, alternative reproductive phenotype, alternative reproductive tactic, diet, major, minor, reproduction, sexual selection

## Abstract

Morphological structures used as weapons in male–male competition are not only costly to develop but are also probably costly to maintain during adulthood. Therefore, having weapons could reduce the energy available for other fitness‐enhancing actions, such as post‐copulatory investment. We tested the hypothesis that armed males make lower post‐copulatory investments than unarmed males, and that this difference will be most pronounced under food‐limited conditions. We performed two experiments using the male‐dimorphic bulb mite *Rhizoglyphus robini*, in which males are either armed “fighters” or unarmed “scramblers.” Firstly, we tested whether fighters and scramblers differed in their reproductive output after being starved or fed for 1 or 2 weeks. Secondly, we measured the reproductive output of scramblers and fighters (starved or fed) after one, two or three consecutive matings. Scramblers sired more offspring than fighters after 1 week, but scramblers and fighters only sired a few offspring after 2 weeks. Scramblers also sired more offspring than fighters at the first mating, and males rarely sired offspring after consecutive matings. Contrary to our hypothesis, the fecundity of starved and fed males did not differ. The higher reproductive output of scramblers suggests that, regardless of nutritional state, scramblers make larger post‐copulatory investments than fighters. Alternatively, (cryptic) female choice generally favours scramblers. Why the morphs differed in their reproductive output is unclear. Neither morph performed well relatively late in life or after multiple matings. It remains to be investigated to what extent the apparent scrambler advantage contributes to the maintenance and evolution of male morph expression.

## 1 INTRODUCTION

Trade‐offs play a vital role in shaping the life history of individuals when the expression of one trait impedes the functionality of another, or when different traits depend on the same, limited resource (Smallegange, 2016; Stearns, 1992). A trade‐off that has recently received attention is that between precopulatory attributes, such as armaments and ornaments, and post‐copulatory attributes, such as sperm number or size (Evans & García‐González, 2016; Parker, Lessells, & Simmons, 2013; Simmons, Lüpold, & Fitzpatrick, 2017). Especially, when competition over access to mates and fertilization of ova is strong and there is little variance between individuals in resource acquisition, males may be restricted to allocating resources to either pre‐ or post‐copulatory attributes (de Jong, 1993; Lüpold, Tomkins, Simmons, & Fitzpatrick, 2014; Parker et al., 2013; Van Noordwijk & de Jong, 1986).

Precopulatory attributes increase male mating opportunities by increasing the likelihood of acquiring mates through male–male competition (fighting or display) or mate choice (Darwin, 1871; Emlen, 2008). Post‐copulatory attributes increase the chances that a male will reproduce by increasing the likelihood of successfully fertilizing ova when a male does mate. A male may become more likely to fertilize ova by transferring more competitive sperm if the female has mated with another male (i.e. in sperm competition [Parker & Pizzari, 2010]) or by favourably biasing cryptic female choice by transferring nutritive or female hormone‐like substances to the female (Eberhard, 1997; Poiani, 2006; Vahed, 1998). Alternatively, males may intentionally or unintentionally manipulate female (remating) behaviour (Parker, 2006) by transferring harmful components (Johnstone & Keller, 2000) or injuring the female during mating (Lange, Reinhardt, Michiels, & Anthes, 2013). Both pre‐ and post‐copulatory attributes can be costly to produce for males. The costs of precopulatory attributes, such as morphological structures that act as weapons, probably remain high after they have been developed, because they can impede efficient locomotion (e.g. Allen & Levinton, 2007; Basolo & Alcaraz, 2003; Goyens, Dirckx, & Aerts, 2015; López & Martín, 2002; Wilson, James, Bywater, & Seebacher, 2009), require large, energy consuming muscles to operate (e.g. Joseph, Emberts, Sasson, & Miller, 2017; Marden, 1989) and/or increase a male's body volume, which increases somatic maintenance costs (Emlen, 2008; Kooijman & Metz, 1983; Parker, 1983). Post‐copulatory attributes such as nuptial gifts (e.g. Perry & Tse, 2013), seminal fluid (Poiani, 2006) and sperm cells (Lüpold et al., 2016; Pitnick, Markow, & Spicer, 1995; Thomsen et al., 2006) can be metabolically expensive to produce. In addition, there may be a locomotive cost of producing post‐copulatory attributes, as testes can take up a large proportion of a male's body mass, for example up to 13.8% in the bush cricket *Platycleis affinis* (Fieber, 1853) (Vahed, Parker, & Gilbert, 2011). The costs of investing in pre‐ or post‐copulatory attributes should give rise to trade‐offs between the attributes if insufficient resources are available.

The costs associated with the possession of precopulatory attributes could limit investment in post‐copulatory attributes. This trade‐off is exemplified by the leaf‐footed cactus bug, *Narnia femorata* (Stål, 1870), which, after autotomizing its weaponized legs during development, is able to grow testes before maturity that are larger than those of nonautotomized (control) males (Joseph et al., 2017). This suggests that the autotomy of its weapons freed up resources that could be invested elsewhere (Joseph et al., 2017). Similarly, the ablation of genital precursor cells in juvenile male horned scarab beetles *Onthophagus taurus* (Schreber, 1759) results in the growth of larger horns compared to unablated males (Moczek & Nijhout, 2004). Such studies indicate that the investment costs of pre‐ and post‐copulatory attributes reciprocally limit their expression. In the leaf‐footed cactus bug and the horned scarab beetle, the trade‐off between investing in pre‐ and post‐copulatory attributes occurs *prior* to maturation (Joseph et al., 2017; Moczek & Nijhout, 2004). The question that we ask here is as follows: Do costs associated with having precopulatory attributes affect post‐copulatory energy budgets and reproductive investment *post* maturation, that is during the adult stage?

In some male‐dimorphic species, during ontogeny, males either do or do not develop precopulatory attributes such as the weaponry used in male–male competition (Oliveira, Taborsky, & Brockmann, 2008). Because of this discrete difference between males, these species are ideal study systems with which to investigate trade‐offs between pre‐ and post‐copulatory attributes and their relative contributions to reproductive success. If males of male‐dimorphic species are in good condition during ontogeny (body size is a commonly used proxy), they are able to develop large weapons that they can use to monopolize females (Oliveira et al., 2008; Tomkins & Hazel, 2007). Males that are in poor condition during ontogeny may still be able to grow small weapons, but they would not be able to compete against males with larger weapons (e.g. Moczek & Emlen, 2000; Tomkins & Hazel, 2007). Instead, males in poor condition often do not grow weapons during ontogeny and adopt alternative reproductive tactics (Tomkins & Hazel, 2007). In some cases, pre‐ and post‐copulatory investments are positively correlated (for a list of examples see Evans & García‐González, 2016), but males that invest highly in both traits may suffer other costs, such as early reproductive senescence (e.g. Preston, Jalme, Hingrat, Lacroix, & Sorci, 2011). In some species, unarmed males invest more in post‐copulatory attributes than armed males (Parker et al., 2013); for example, unarmed males may produce more sperm cells to increase the probability of fertilizing ova in sperm competition when they do get to mate (e.g. Locatello, Pilastro, Deana, Zarpellon, & Rasotto, 2007). Crucially, in some male‐dimorphic species, males are unable to shed weapons after developing them (Oliveira et al., 2008) and are bound to the weapons’ maintenance costs. As a result of the limitations imposed by the obligatory costs of weapons and the costs of investing in post‐copulatory attributes, we predict that a trade‐off between pre‐ and post‐copulatory attributes is more likely under extended periods of limited food availability (e.g. Droney, 1998; Gage & Cook, 1994; Simmons, 2012).

In this study, we tested the hypothesis that during the adult stage, costs associated with having precopulatory weaponry adversely affect post‐copulatory energy budgets and investment, and consequently reproductive output, particularly under food‐limited conditions. To test our hypothesis, we used the male‐dimorphic bulb mite *Rhizoglyphus robini* (Claparède, 1869). In *R. robini*, adult males differ in their third leg pair: the armed “fighter” has an enlarged third leg pair with a sharp end that functions as a weapon to kill rivals. The third leg pair of the unarmed “scrambler” is not enlarged, and scramblers cannot kill competitors (Radwan, Czyż, Konior, & Kołodziejczyk, 2000). Male morph expression in *R. robini* is partly heritable (Radwan, 2003; Smallegange & Coulson, 2011), but because male morph determination in *R. robini* follows a conditional strategy (Tomkins & Hazel, 2007), it is also to a large extent environmentally determined: only relatively large male nymphs become fighters, as only they have accumulated a sufficient amount of resources to develop fighter legs (Smallegange, 2011a). Fighters have several fitness benefits over scramblers. Fighters can kill other mites, which allows them to eliminate rivals, monopolize access to females and even obtain additional resources through cannibalism (Radwan & Klimas, 2001; Radwan et al., 2000; Smallegange & Deere, 2014). Scramblers have a shorter maturation time than fighters, so scramblers can mate earlier in life than fighters from the same cohort (Smallegange, 2011b). Scramblers also live longer than fighters (Radwan & Bogacz, 2000); however, the reproductive output of both morphs decreases with age and the number of previous mates (Radwan & Bogacz, 2000); hence, the reproductive benefit of an increased longevity is questionable. There is no evidence that either male morph differentially invests in post‐copulatory attributes such as sperm competition (Radwan, 1997); however, Van den Beuken and Smallegange (2018b) found that, after allowing 1 day of feeding, males of both morphs sired more offspring than starved males. These results suggest that males transferred resources to their mate which increased offspring production, for example a nuptial gift. Because having precopulatory attributes and producing post‐copulatory attributes are probably costly during the adult stage, we surmise that there is a trade‐off between having weaponry versus being able to invest in post‐copulatory attributes, which affects reproductive output. To test for the existence of this trade‐off, we performed two experiments: a “single mating” and a “multiple matings” experiment. In both experiments, we used female reproductive output as a proxy for male post‐copulatory investment.

In the single mating experiment, we assessed if male morphs differed in their investment into post‐copulatory attributes that would increase female fecundity (e.g. nuptial gifts). As a proxy of the investments in these post‐copulatory attributes, we tested whether there was a difference in reproductive output between virgin females that were mated with a fighter (with precopulatory attributes) and those that were mated with a scrambler (without precopulatory attributes), and whether this result was affected if males had been starved or fed, for 1 or 2 weeks. Females can be more susceptible to nutritional contributions when they are starved (e.g. Immonen, Hoikkala, Kazem, & Ritchie, 2009). Therefore, we starved females in the single mating experiment.

It is possible that, rather than investing in attributes that increase female fecundity (single mating experiment), a male morph invests more resources into multiple copulations (multiple matings experiment). In the multiple matings experiment, we tested whether there was a difference in reproductive output between virgin females that were mated with a fighter, and those that were mated with a scrambler after the male's first, second and third mating, separated by 2‐hr intervals. Males were starved or fed for 6 days prior to the first mating. In this experiment, our focus lies on the ability of males to invest resources in multiple matings, not on producing nuptial gifts for multiple matings. Therefore, we fed females in order for the females’ nutritional state not to limit the potential reproductive output of males.

Males in both experiments were starved because we assumed that the constraints on investment in reproduction imposed by precopulatory attributes are most pronounced in energy limited, that is starved, circumstances. In both experiments, we included a control treatment in which males were provided *ad libitum* access to food. In the single mating experiment, this was conducted to control for any age effects in males (e.g. reproductive senescence [Radwan & Bogacz, 2000]), and in the multiple matings experiment, it was conducted to test whether investment in multiple reproductive events is dependent on male nutritional state.

We hypothesized that (a) scramblers would sire more offspring than fighters in both experiments, and that the differences between the two morphs would be most pronounced in starved males; (b) fed males would sire more offspring than starved males in both experiments; and (c) the number of sired offspring would decrease for both morphs, but particularly for fighters, with increasing male age (single mating experiment) and number of consecutive matings (multiple matings experiment).

## 2 MATERIALS AND METHODS

### 2.1 The bulb mite *R. robini*

The subterraneous bulb mite *R. robini* is a pest of a wide array of agriculturally important plants including garlic, onion, carrot, rye and several ornamental plants and can be found all over the world (Díaz, Okabe, Eckenrode, Villani, & O'Connor, 2000). The bulb mite goes through five or six stages during its development: egg, larva, protonymph, deutonymph (facultative dispersal stage that only occurs under adverse conditions), tritonymph and adult (Baker, 1982). Except for the larval stage, each stage is preceded by a quiescent phase during which the mite is immobile until it moults. Only during the adult stage is the mite's sex or male morph identifiable. The development from egg to adult takes from 11 to 40 days (Smallegange, 2011a) and the adult stage lasts 31–130 days, depending on various environmental factors (Díaz et al., 2000; Gerson, Capua, & Thorens, 1983).

#### 2.1.1 Maintenance of mites

Mites for the stock cultures were collected from flower bulb storage rooms in Anna Paulowna, North Holland (the Netherlands), in 2010 (50 randomly selected founding individuals for each of four, periodically mixed stock cultures). The stock cultures had been maintained at the University of Amsterdam (the Netherlands) for just over 2 years before the start of the experiment and were kept in small plastic tubs (l × w × h: 8 × 8 × 3 cm) that were two‐thirds filled with plaster of Paris. Water drops and yeast granules (Bruggeman instant yeast) were added on top of the plaster to provide the mites with food and water. A sixth of the substratum was scraped clean of yeast and detritus twice a week, and several drops of water and yeast granules were placed in the scraped area. In order to allow ventilation and water evaporation, a square hole (approximately l × w: 2 × 2 cm) was cut into the centre of the lid and covered with fine mesh to prevent the mites from escaping.

For the duration of the experiment, mites were kept in plastic “individual” tubes (h × d: 50 × 16 mm), either individually or in pairs (see Experimental setup). The tubes were two‐thirds filled with a mixture of plaster of Paris and powdered charcoal for a visual contrast between the mites and the substratum. Yeast (if the treatment required it, see Experimental setup) and water (almost a saturating quantity) were replenished once a week for the entire duration of the replicate block. The caps used to close the tubes were punctured to allow ventilation and water evaporation from the tube. The same fine mesh that was used to cover the population tubs was also used between the cap and the tube to prevent the mites from escaping through the hole. Both the stock cultures and the individual tubes were kept at 25°C at >70% relative humidity in an unlit incubator.

#### 2.1.2 Experimental setup

##### Single mating experiment

**Figure 1:**
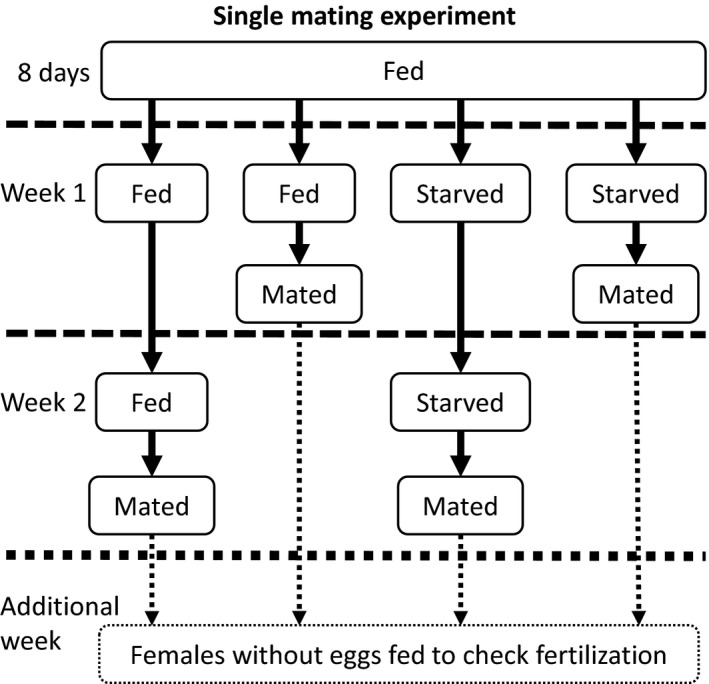
Experimental design of the single mating experiment. All males were fed for the first 8 days after adult emergence. Thereafter, males were either fed or starved for a week and then mated with a standardized, starved, virgin female, or fed or starved for two consecutive weeks and then mated. The female was starved before, during and after mating. After mating, the female was isolated. One week later, the number of eggs laid by the female was counted. Some females that did not lay eggs during this week. To check whether this was because they were not fertilized or lacked nutritional resources, we fed them *ad libitum* food, and counted the number of eggs during this additional week. The described methods were applied in the same way to fighters and scramblers

The single mating experiment comprised three treatments: (a) male morph (MM: fighter or scrambler), (b) male nutritional state (NS: starved or fed; started after an initial 8 days of feeding, see Figure 1) and (c) mating week (MW: week 1 or 2; counted after the initial 8‐day feeding period, see Figure 1). The experiment had a randomized block design in which each replicate block comprised all eight treatment combinations, that is, all pairwise combinations of the three treatments. The response variable was the mean total number of eggs laid by the mate of each male within 1 week of mating. In total, 10 replicate blocks were completed. Each block started 1 week after the previous one.

On the first day of each replicate block, 200 quiescent tritonymphs (sessile moulting stage before the mite enters the adult stage, lasting approximately a day) were collected from the four stock cultures (50 from each). The collected mites were stored individually in tubes without food. The next day, almost all of the collected mites had matured, and only male mites were kept. A dorsal photograph was taken of each of these males (Zeiss Stemi 2000‐C microscope equipped with a Zeiss Axiocam 105 colour camera, 0.63‐5× magnification). Using this image, we measured male idiosoma length (body length minus the chelicerae) over the anteroposterioral axis to the nearest 4.5 μm (uncertainty calculated from the standard deviation of 10 repeated measures) using Zen 2.3 (Blue edition) software. Such a length measurement is a standard proxy for body condition in the bulb mite and is the easiest measurement that can be taken of a mite while reducing measurement error due to mite movement. We selected scrambler and fighters (treatment: male morph) in roughly equal numbers for this experiment (see Supporting Information Table S1.A). After the males were photographed, they were individually housed in tubes with *ad libitum* food in an unlit incubator for 8 days to build up a reserve. All of the males were given a single opportunity to mate with a female over 24 hr: either 1 week after the initial 8‐day feeding period (week 1) or 2 weeks after the initial 8‐day feeding period (week 2) (treatment: mating week, see Figure 1). During these 1 or 2 weeks, males were either continuously fed or starved (treatment: male nutritional state, see Figure 1). Half of the males were starved during these 1 or 2 weeks to assess the rate of reserve depletion. The other half were fed in order to control for age effects on male reproductive output (e.g. reproductive senescence [Radwan & Bogacz, 2000]). Both females and males, including fed males, were starved during the 24‐hr mating period. As male size may have changed after the first measurement because of ageing, feeding or starving, and size may explain differences in female fecundity, we measured the males again before mating. Females were measured only once, after mating. The mating period was set at 24 hr because (a) this should have been more than enough time to complete mating (and mate searching) (Radwan & Siva‐Jothy, 1996), (b) a previous study found that fighters often kill females if left without food for an extended period of time (Van den Beuken & Smallegange, 2018b), and (c) we wanted to assess whether males, particularly starved males, died more often after mating, perhaps as a result of a terminal reproductive investment; however, no male died of such causes.

All of the females were obtained as quiescent tritonymphs from each of the four stock populations 5 days before they were paired with a male. Between 50 and 250 quiescent tritonymphs were collected to obtain females. This number depended on the number of males obtained earlier and the number of overlapping replicate blocks. Because we were interested in differences between the male morphs in siring offspring (e.g. by transferring nuptial gifts), emerging females were starved after maturing and for the duration of the experiment. Seven days after the males and females were separated, the number of eggs produced by the females was counted and used as a proxy for male post‐copulatory investment.

We noticed that a considerable number of these females did not produce eggs. Therefore, from replicate block 3 onwards, we provided non‐egg‐producing females with *ad libitum* yeast for a week after the trial to ascertain whether they had been fertilized (and were fertile). We found that about one‐third of these females did not produce offspring and had probably not been fertilized. As we could not check whether females from blocks 1 and 2 had been fertilized, we omitted females that did not lay eggs during the first week of blocks 1 and 2 (43 of 49 omitted; in one couple the female died). In blocks 3–10, we omitted data points if the male died before the mating period was over, females laid no eggs during either the experimental or feeding week, or if they died before the number of offspring was assessed (74 of 139 were omitted; in five couples the male or female died). One data point was omitted because the female size measurement was missing. The remaining 70 trials were included in the analyses (see Supporting Information Table A1.A for replicates per treatment combinations). Because data of the females that did not lay eggs during the experimental week and were fed afterwards were inherently biased, we did not analyse the effects of any of the treatments on the number of eggs laid by these fed females.

##### Multiple matings experiment

**Figure 2:**
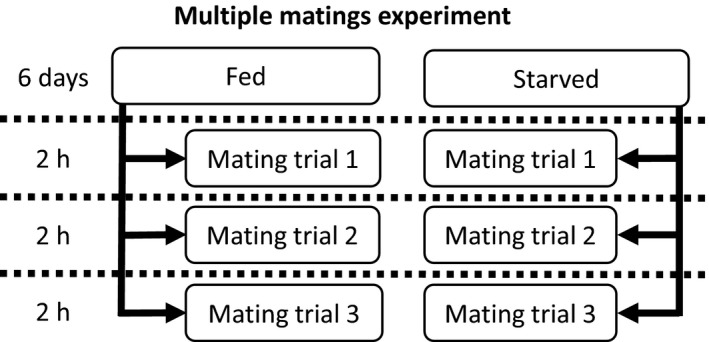
Experimental design of the multiple matings experiment. Males were either continuously fed or starved during the first 6 days after adult emergence. Hereafter, they were mated to a total of three females. Each female was in a tube with the male for 2 hr. The number of eggs laid by the female was counted until the female laid no more eggs for 2 weeks, during which time the female was fed *ad libitum* yeast

This experiment comprised three treatments: (a) male morph (fighter or scrambler), (b) male nutritional state (starved or fed) and (c) mating trial (first, second or third trial) (Figure 2). All of the mites were obtained as quiescent tritonymphs from the stock populations. The quiescent tritonymphs and the adults that emerged from the tritonymphs the next day were stored individually in tubes. In contrast to the single mating experiment, in which all of the females were starved with the exception of the post‐trial egg‐laying week, all of the adult females that emerged were supplied with *ad libitum* yeast. We fed all of the females so that their egg production would not be constrained, as the goal of this experiment was to investigate whether males of different morphs or nutritional states invest differentially in consecutive matings. Adult scramblers and fighters (treatment: male morph) were randomly assigned a “fed” or “starved” treatment (approximately half of the scramblers and half of the fighters, see Supporting Information Table S1.B) in which they were given *ad libitum* access to food or no food, respectively (treatment: nutritional state, see Figure 2). Six days after the adult males had emerged, they were transferred to an individual tube without food. Here, each male was subjected to three consecutive mating trials. During each 2‐hr mating trial, each male was paired with a different virgin female (treatment: mating trial, see Figure 2). Bulb mites are known to copulate for approximately 20 min (Radwan & Siva‐Jothy, 1996), so 2 hr should be more than enough for at least one successful copulation (cf. Smallegange, Thorne, & Charalambous, 2012). We recorded whether copulation took place. After mating, the males were discarded and the females were transferred back to their individual tubes where they had access to *ad libitum* yeast and could lay eggs. Total egg production was then recorded until a female laid no eggs for two consecutive weeks, after which point we assumed it would not produce any more eggs. To prevent hatched individuals from affecting the female oviposition rate, females were transferred to a new tube every week. A total of 91 males were paired with three females each. Data were omitted if females died before finishing the 2‐week period during which they laid no eggs (omitted data points: mating trial 1, 4; mating trial 2, 2; and mating trial 3, 0). Mating trial 1 data were only used if the male copulated with the female (*n = *37), mating trial 2 data were only used if the male sired offspring during mating trial 1 and copulated during mating trial 2 (*n* = 4), and mating trial 3 data were only used if the male sired offspring during the previous two mating trials and copulated during mating trial 3 (*n* = 0). Given the low number of replicates for matings trials 2 and 3, we could only analyse data for mating trial 1. See also Supporting Information Table S1.B for replicates per treatment combinations and the number of (successful) matings.

### 2.2 Statistical analyses

For the single mating experiment, we used a generalized linear model (GLM) with a Poisson error distribution (checked for overdispersion) to analyse how the mean number of eggs laid by a female within a week of mating was affected by the main effects male morph (MM), nutritional state (NS) and mating week (MW), as well as a three‐way interaction (MM × NS × MW) and three two‐way interactions between these three treatments (MM × NS, MM × MW and NS × MW). As covariates, we included male length after adult emergence (“male length 1,” ML1), male length before mating (“male length 2,” ML2), female length after mating (“female length,” FL) and the stock population from which each male (PM) and female (PF) was obtained. A linear model was fitted to test whether male size before mating was related to male morph.

For the multiple matings experiment, we tested for the effects of male morph (MM), nutritional state (NS) and their two‐way interaction (MM × NS) on the number of eggs produced by each female using a GLM with a quasi‐Poisson error distribution.

To select the best statistical model for both experiments, we used a model simplification procedure that produced a reduced model from the full model by first removing the least significant term of the highest order. The difference in deviance between the reduced and full model was then tested using a likelihood ratio test, in which the difference in deviance between the two models followed a chi‐squared distribution. If this test indicated a significant increase in deviance (*p* < 0.05), then the previously removed term was retained in the fitted model; if the increase was not significant, the term was removed from the model (Crawley, 2007). These steps were repeated until only terms remained of which the removal led to a significant increase in deviance (see Supporting Information Tables S2 and S3 for the model simplification steps of the single mating and multiple matings experiments, respectively). In the Section 3, we present the parameter estimates ($\hat{e}$) of each statistically significant term in the best‐fitting minimal model. Contrasts amongst the treatments in significant interactions or main effects were obtained through general linear hypothesis testing. All of the analyses were performed using R version 3.3.2 (R Core Team, 2017) integrated in RStudio version 1.1.383 (RStudio Team, 2017). We used the R packages “stats” for GLM analyses (R Core Team, 2017), “emmeans” (Lenth, 2018) and “multcomp” (Hothorn, Bretz, & Westfall, 2008) for general linear hypothesis testing and “ggplot2” for producing figures (Wickham, 2016).

## 3 RESULTS

### 3.1 Single mating experiment

**Figure 3:**
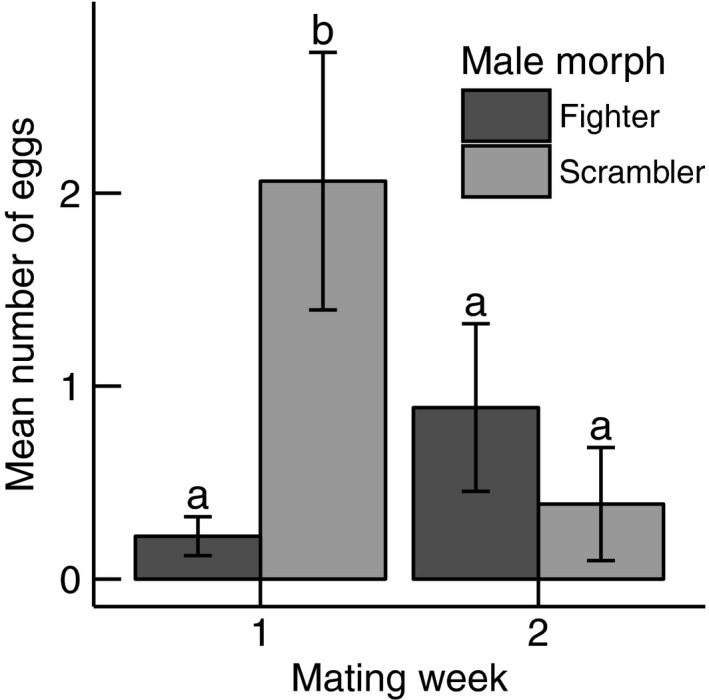
Effect of the interaction between male morph and mating week on the mean number of eggs laid by the female (single mating experiment). This graph uses the pooled data of starved and fed males, as the male nutritional state had no statistically significant effect on female fecundity. Untransformed data are shown. Statistically significant differences between treatment combinations are indicated by different letters above the error bars (general linear hypothesis testing after a generalized linear model: *p* < 0.05). Vertical lines are standard errors

We found a significant effect of the interaction between male morph and mating week on the number of eggs produced by a female (MM × MW: $\chi_{1}^{2}$ = 4.575, *p* = 0.032). Specifically, females mated to scramblers in mating week 1 produced more eggs than those mated to scramblers in mating week 2, and those mated to fighters in either mating week. The latter three treatment groups did not differ amongst each other (see Figure 3, contrasts between treatments tested using general linear hypothesis testing; *p*‐values and estimates are given in Supporting Information Table S4). There was no significant effect of the male's nutritional state on the mean number of eggs produced by the female (NS: $\chi_{1}^{2}$ = 1.218, *p* = 0.270). Furthermore, the mean number of eggs laid by females was negatively correlated with male body length just before mating (ML2: $\chi_{1}^{2}$ = 14.575, *p* < 0.001) but positively correlated with female body length (FL: $\chi_{1}^{2}$ = 30.175, *p* < 0.001). Which of the four stock populations, a female was obtained from significantly affected the mean total number of eggs produced by a female (PF: $\chi_{3}^{2}$ = 10.770, *p* = 0.013; for means, standard errors and general linear hypothesis testing see Supporting Information Table S5). A linear model found no significant correlation between male morph and male body length before mating (MM [scrambler]: *t* = 0.959, *p* = 0.341, *R* ^2^ = 0.013). The significant results are summarized in Table 1.

**Table 1:** Interactions and main effects that remained in the final model of the single mating experiment. For the full model simplification steps and additional model coefficients, see Supporting Information Table S2

	Term	χ^2^ (*df*)	*p*‐Value	Details
1	MM × MW	4.575 (1)	0.032	See Figure 3
2	ML2	3.481 (1)	<0.001	$\hat{e}$ = −0.023, *SE* = 0.007, *z* = −3.481
3	FL	30.175 (1)	<0.001	$\hat{e}$ = 0.014, *SE* = 0.003, *z* = 4.936
4	PF	10.770 (3)	0.013	See Supporting Information Table S5

Notes

FL: female length after mating; MM: male morph; MW: mating week; ML2: male length before mating.

The stock population number from which the female was obtained (PF). Estimates ($\hat{e}$) are included for significant main effects with one level.

### 3.2 Multiple matings experiment

**Figure 4:**
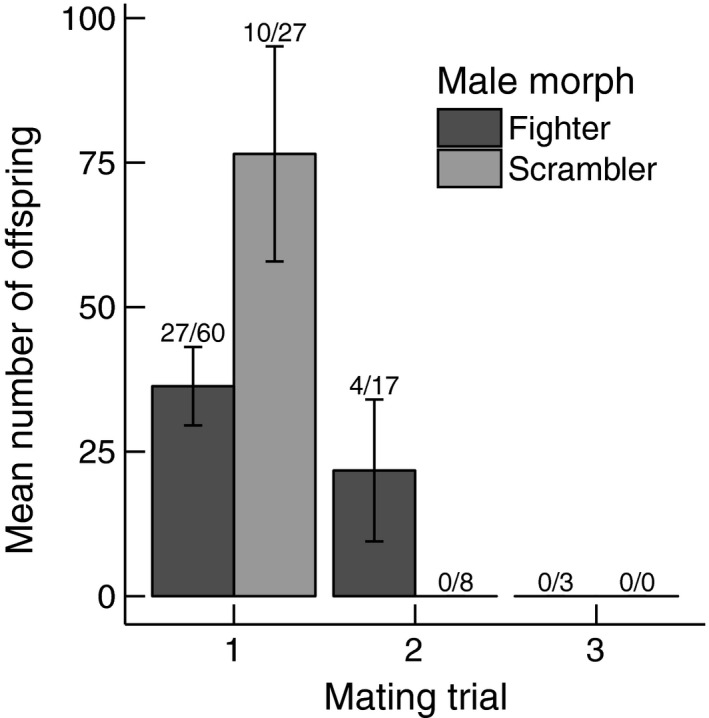
Number of offspring sired by scramblers or fighters after the first, second or third mating trial in the multiple matings experiment. Numbers above the error bars indicate the number of males that mated with a female out of the number of males that were paired with a female (mating trial 1), the number of males that mated with a female out of the number of males that sired offspring with female 1 (mating trial 2) or the number of males that mated with a female out of the number of males that sired offspring with females 1 and 2 (mating trial 3). Vertical lines are standard errors

As in the single mating experiment, scramblers sired significantly more offspring than fighters in mating trial 1 (MM (scrambler): $\chi_{1}^{2}$ = 226.260, *p* = 0.013; Figure 4). We found no significant interaction between male morph and nutritional state (MM × NS: $\chi_{1}^{2}$ = 7.652, *p* = 0.648) and no significant effect of nutritional state (NS: $\chi_{1}^{2}$ = 95.651, *p* = 0.104) (for model simplification steps and parameter estimates see Supporting Information Table S3). Only four males that mated with the first female also mated with the second female, these males were all fighters. No male mated with all three females (Figure 4). Given the low number of mating males (see Supporting Information Table S1.B), we did not analyse the effects of male morph or nutritional state on offspring production in mating trials 2 and 3.

## 4 DISCUSSION

We investigated whether there is a trade‐off in adult males between the possession of *pre*copulatory attributes and the opportunity for *post*‐copulatory investment to increase offspring production in the male‐dimorphic bulb mite *R. robini*. We found that (a) scramblers sired more offspring than fighters, (b) this was regardless of the male's nutritional state, and (c) young scramblers sired more offspring than old scramblers. There was no difference in the number of offspring sired between young and old fighters, and reproductive output declined sharply after the first mating.

Scramblers in both the single mating and multiple matings experiments sired more offspring than fighters. In the single mating experiment, scramblers sired more offspring than fighters after 1 week, but there was no difference after 2 weeks. In the multiple matings experiment, scramblers sired more offspring than fighters after the first mating, but not after the following matings. This could be a result of (cryptic) female choice as females could prefer to invest in reproducing with scramblers because female offspring of scramblers has a higher fitness than the female offspring of fighters (Stuglik, Babik, Prokop, & Radwan, 2014). In other words: the level of intralocus sexual conflict may be lower between females and scramblers than between females and fighters (Bonduriansky & Chenoweth, 2009). Other studies support the possibility of a lower intralocus sexual conflict between females and scramblers as bidirectional selection for male morph expression yields higher fitness daughters in scrambler lines than in fighter lines (Plesnar‐Bielak, Skwierzyńska, Miler, & Radwan, 2014; Van den Beuken & Smallegange, 2018a). Alternatively, scramblers transfer an oviposition‐stimulating compound to females to increase the males’ reproductive output. To the best of our knowledge, there is no empirical evidence that scramblers transfer an oviposition‐stimulating compound to females, but it does occur in insects in which males produce substances during copulation that can induce ovulation and oviposition in females (Cordero, 1995; Poiani, 2006). Compounds that induce ovulation or oviposition can be costly to produce (Cordero, 1995), so it is possible that the metabolic costs of fighter legs limit the amount of energy available to synthesize the compound. Therefore, the higher reproductive output of scramblers could be explained by (cryptic) female choice favouring scramblers or by oviposition‐stimulating compounds that are (temporarily) transferred by scramblers but not fighters.

We did not find any effect of nutritional state on the reproductive output of males of different morphs, neither did we find that scramblers performed better than fighters under starved conditions. This does not support our hypothesis or the results obtained by Van den Beuken and Smallegange (2018b), who, in a similar experiment, found that starved females mated to “fed” males produced more offspring than starved females mated to “starved” males. Importantly, after reaching maturity, “fed” males were fed for a single day and “starved” males were always starved in the study by Van den Beuken and Smallegange (2018b). In the present study, we fed both “starved” and “fed” males in the single mating experiment for 8 days prior to starting the experiment. Hence, the reserves built up during this period may have (largely) negated the effects of starvation or feeding afterwards. Although we did not feed “starved” males in the multiple matings experiment from maturity onwards (as in Van den Beuken & Smallegange, 2018b), the opportunity to mate was considerably shorter (3 × 2 hr versus 10 days), which may not have been sufficient time for fed males to sire more offspring than starved males. Van den Beuken and Smallegange (2018b) proposed that males increased their fecundity by transferring nutritious nuptial gifts to females. If this were the case, in our experiment, we would have expected that a nutritious nuptial gift would result in a certain, more‐or‐less fixed number of eggs produced, regardless of female nutritional state (possibly some of the gift could be used for the starved female's metabolism, rather than for eggs, see Voigt, Kretzschmar, Speakman, & Lehmann, 2008). Instead, we found that the egg production of *fed* females mated to scramblers or fighters was several orders of magnitude higher than that of *starved* females mated to scramblers or fighters. As we discussed in the previous paragraph, it is therefore probable that other mechanisms underlie females’ increased egg production when mated to scramblers, such as (cryptic) female choice for scramblers, or an oviposition‐stimulating compound that is transferred by scramblers (which may bias cryptic female choice).

In the single mating experiment, the scrambler effect on the reproductive output of females was only evident in the first mating week, but not in the second mating week. This could have been an effect of reproductive senescence (e.g. Bonduriansky & Brassil, 2002), which affects reproductive output in male bulb mites (Radwan & Bogacz, 2000). Alternatively, scramblers may invest more in reproduction during early adulthood, and adaptively decrease their investment later in life, for example in order to prolong their lifespan (Williams, 1966; e.g. Cotter, Ward, & Kilner, 2011). In the multiple matings experiment, no additional offspring were produced if a scrambler mated more than once, and only 10% of fighters that mated with the first female sired offspring after the second mating. This may be a mating strategy that allocates maximum resources to the first mating opportunity (Wedell, Gage, & Parker, 2002). It does appear that the reproductive benefit of scramblers over fighters is context‐dependent and may be short‐lived.

Regardless of what the underlying mechanisms are, our results reveal a direct link between the presence or absence of precopulatory attributes and reproductive output in the absence of sperm competition. Theory predicts that with increasing population density, the number of mates each female copulates with will increase, so the benefits of precopulatory attributes (to increase the chance of obtaining females) decrease and the benefits of post‐copulatory attributes (to increase the chance of producing offspring when mating) increase (McCullough, Buzatto, & Simmons, 2018; Parker & Birkhead, 2013; Parker & Pizzari, 2010). It follows that the reproductive benefit of investing in post‐copulatory attributes rather than precopulatory attributes also increases with increasing population density (McCullough et al., 2018; Parker & Birkhead, 2013; Parker & Pizzari, 2010). Indeed, under low food conditions, bulb mite males are mostly fighters (Smallegange, Fernandes, & Croll, 2018), but under strong density‐dependent conditions, male morph expression is biased towards scramblers (Smallegange & Deere, 2014). However, these results could also be explained by the costly expression of the fighter phenotype under a high population density (and hence limited food). Therefore, we need to unravel how fighter and scrambler fitnesses depend upon population density in order to understand how *R. robini* male morph expression varies over time. Our results highlight the complexity of how different processes affect trade‐offs between pre‐ and post‐copulatory attributes and the expression of alternative morphs.

## CONFLICT OF INTEREST

The authors have no conflict of interest to declare.

## Data Availability

Data deposited at Figshare: https://doi.org/10.6084/m9.figshare.7378304
