# Supplementary material for: Costs of weaponry: Unarmed males sire more offspring than armed males in a male‐dimorphic mite
Source: J Evol Biol. 2018 Dec 3;32(2):153–62. doi: 10.1111/jeb.13402 (PMC7379726; doi:10.1111/jeb.13402)
Supplement: Supplementary file 1 [file JEB-32-153-s001.docx]

# Supporting Information S1

Supporting information to:

**Costs of weaponry: unarmed males sire more offspring than armed males in a male-dimorphic mite**

We performed two experiments: the ‘single mating’ and the ‘multiple matings’ experiment. In both experiments, we manipulated the nutritional state of males. In the single mating experiment, we varied the amount of time males spent being fed or starved before mating with one female. In the multiple matings experiment the amount of time spent being fed or starved did not differ, but males mated with three consecutive females.

In the single mating experiment, all males were fed for eight days after which they were either starved or fed *ad libitum* until they were mated with a female. All males were singly mated to a virgin female once and for one day: half of the males were mated one week after the initial eight days ended (mating week 1), the other half of the males was mated two weeks after the initial eight days (mating week 2) (number of replicates per treatment group in Table S1.A). As a response variable we counted the number of eggs laid by the female one week after the male and female finished mating. We used a Generalized Linear Model (GLM) with Poisson error distribution to analyse our results. In Table S2 we show what model simplification steps we have taken to go from the full GLM model to the final model. Our results show, amongst others, that the mean number of eggs produced by females is dependent on an interaction between the male’s morph and mating week. We used general linear hypothesis testing to test for statistically significant treatment factors within this interaction; the pairwise results of these tests are shown in Table S4. We found that the stock population from which we obtained the female affected the number of eggs she laid. Table S5 shows the mean number of eggs laid by females obtained from each of the four stock populations, as well as the contrasts between populations calculated using general linear hypothesis testing.

In the multiple matings experiment we starved or fed adult male fighters or scramblers for six days after adult emergence before pairing them with three consecutive females (number of replicates per treatment group in Table S1.B). Each male was paired with a female for two hours during which we checked whether they mated or not. The number of eggs produced by each mated female was our response variable. We used a GLM with quasi-Poisson error distribution to analyse our results. As with the single mating experiment we used model simplification steps to reduce the full model to the final model, the steps are shown in Table S3.

## Tables

| Table S1. Replicate number per treatment combination for the single mating (A) and multiple matings experiment (B). Table A shows the number of pairs that successfully mated (i.e. produced eggs during the trial or during the following feeding period) / the number of pairs that were checked for successful mating. Table B shows the number of males that were *paired* with a female / the number of males that *copulated* with the female / the number of males that *sired offspring* with the female / the number of males that *sired offspring with this female and the previous female(s)* (only trials 2 and 3). In both datasets, we omitted data points if the female (single mating experiment) or if one of the previous females (multiple matings experiment) died before the final assessment of her reproductive output. The replicate numbers of data used for the analyses are printed in bold. | | | | | | | | | | |
| --- | --- | --- | --- | --- | --- | --- | --- | --- | --- | --- |
| **A**  **Mating week** | **Fighter** | | | **Scrambler** | | | | | |  |
|  | **Starved** | **Fed** | | **Starved** | | | **Fed** | | | |
| 1 | **11** / 16 | **7** / 9 | | **7** / 9 | | | **9** / 12 | | | |
| 2 | **11** / 13 | **7** / 9 | | **8** / 12 | | | **10** / 14 | | | |
|  |  | |  | |  | | |  |  |  |
| **B**  **Mating trial** | **Fighter** | | | | | **Scrambler** | | | | |
|  | **Starved** | **Fed** | | **Starved** | | | **Fed** | | | |
| 1 | 32 / **17** / 10 / - | 28 / **10** / 7 / - | | 13 / **4** / 3 / - | | | 14 / **6** / 5 / - | | | |
| 2 | 31 / 4 / 2 / **2** | 28 / 3 / 1 / **1** | | 13 / 0 / 0 / **0** | | | 14 / 1 / 1 / **0** | | | |
| 3 | 31 / 1 / 0 / **0** | 28 / 0 / 0 / **0** | | 13 / 0 / 0 / **0** | | | 14 / 2 / 0 / **0** | | | |

| Table S2. Single mating experiment: model simplification steps used for the analysis of the effect of male morph, nutritional state and mating week on the mean total number of eggs laid by a female. We used a GLM with Poisson error distribution to test for the effects on the mean number of eggs laid by females during the week after mating of the main effects male morph (MM), nutritional state (NS) and mating week (MW), the three two-way interactions between these three treatments (MM × NS, MM × MW and NS × MW), the three-way interaction (MM × NS × MW) as well as the covariates male length after emergence (male length 1: ML1), male length before mating (male length 2: ML2), female length after mating (female length: FL) and the stock population number from which each male (PM) and the female (PF) was obtained. From this full model, we removed the least significant term (starting with the highest order interaction), in underscored font below, producing a reduced model. We tested for statistically significant increases in deviance between the full and reduced models using a likelihood ratio test compared to a chi-squared distribution. If the increase in deviance was significant (i.e. *χ*²_1_: *P*-value < 0.05, printed in bold), then the fitted model in the next step will be the same as the previous step; else (*χ*²_1_: *P*-value>0.05) the reduced model becomes the fitted model of the next step. Here, the next least significant term, starting with the highest order interaction, was removed from the fitted model to create a new reduced model. Terms were removed until only terms remained in the final model of which the removal would lead to a significantly lower deviance. At the bottom of the table we present the parameter estimates of each main effect and interaction in the final model. |
| --- |
| \| Full model: GLM: Mean number of eggs laid by female ~ MM ⋅ NS ⋅ MW + ML1 + ML2 + FL + PM + PF. Poisson error structure. *N* = 70. \| \| \| \| \| \| --- \| --- \| --- \| --- \| --- \| \| **Step #** \| **Fitted model** \| **Reduced model** \| ***χ*²_1_** \| ***P*-value** \| \| 1 \| MM ⋅ NS ⋅ MW + ML1 + ML2 + FL + PM + PF \| MM ⋅ NS + MM ⋅ MW + NS ⋅ MW + ML1 + ML2 + FL + PM + PF \| 2.648 \| 0.104 \| \| 2 \| MM ⋅ NS + MM ⋅ MW + NS ⋅ MW + ML1 + ML2 + FL + PM + PF \| MM ⋅ MW + NS ⋅ MW + ML1 + ML2 + FL + PM + PF \| 1.933 \| 0.164 \| \| 3 \| MM ⋅ MW + NS ⋅ MW + ML1 + ML2 + FL + PM + PF \| MM ⋅ MW + NS + ML1 + ML2 + FL + PM + PF \| 0.876 \| 0.349 \| \| 4 \| MM ⋅ MW + NS + ML1 + ML2 + FL + PM + PF \| MM + MW + NS + ML1 + ML2 + FL + PM + PF \| 4.575 \| **0.****032** \| \| 5 \| MM ⋅ MW + NS + ML1 + ML2 + FL + PM + PF \| MM ⋅ MW + NS + ML2 + FL + PM + PF \| 0.123 \| 0.726 \| \| 6 \| MM ⋅ MW + NS + ML2 + FL + PM + PF \| MM ⋅ MW + ML2 + FL + PM + PF \| 1.218 \| 0.270 \| \| 7 \| MM ⋅ MW + ML2 + FL + PM + PF \| MM ⋅ MW + ML2 + FL + PF \| 1.351 \| 0.717 \| \| 8 \| MM ⋅ MW + ML2 + FL + PF \| MM ⋅ MW + ML2 + FL \| 10.770 \| **0.013** \| \| 9 \| MM ⋅ MW + ML2 + FL + PF \| MM ⋅ MW + FL + PF \| 14.575 \| **< 0.001** \| \| 10 \| MM ⋅ MW + ML2 + FL + PF \| MM ⋅ MW + ML2 + PF \| 30.175 \| **< 0.001** \| \| Final model: Mean number of eggs laid by female ~ MM ⋅ MW + ML2 + FL + PF \| \| \| \| \|  \| **Final model coefficients [relative to intercept]** \| ***z*** \| **Estimate (*ê*)** \| **Std. Error** \| \| --- \| --- \| --- \| --- \| \| Intercept [fighter, mating week 1, female stock pop. 1] \| 0.731 \| 2.582 \| 3.532 \| \| Male morph [scrambler] (MM) \| 2.806 \| 1.575 \| 0.561 \| \| Mating week [2] (MW) \| 1.425 \| 0.836 \| 0.587 \| \| Male length 2 (ML2) \| −3.481 \| −0.023 \| 0.007 \| \| Female length (FL) \| 4.936 \| 0.014 \| 0.003 \| \| Female stock population number [2] (FP) \| 0.009 \| 0.004 \| 0.387 \| \| Female stock population number [3] (FP) \| −1.181 \| −0.452 \| 0.383 \| \| Female stock population number [4] (FP) \| −2.362 \| −1.774 \| 0.751 \| \| Male morph [scrambler] × Mating week [2] (MM × MW) \| −1.864 \| −1.491 \| 0.800 \| |

| Table S3. Multiple matings experiment: model simplification steps used for the analysis of the effect of male morph and nutritional on the number of eggs laid by the first female. We used a GLM with quasi-Poisson error distribution to test for the effects on the mean total number of eggs laid after mating trial 1 of the main effects male morph (MM) and nutritional state (NS) and the two-way interaction between these two main effects (MM × NS). Model simplification steps were performed as described in the legend of Table S2. Underlined main factors or interactions were removed from the full model, *P*-values indicating a statistically significant increase in deviance (i.e. *χ*²_1_: *P*-value < 0.05) are printed in bold. At the bottom of the table we present the parameter estimates of the remaining main effect in the final model. |
| --- |
| \| Full model: GLM: Mean number of eggs per female ~ MM ⋅ NS. Quasi-Poisson error structure. *N* = 37. \| \| \| \| \| \| --- \| --- \| --- \| --- \| --- \| \| **Step #** \| **Fitted model** \| **Reduced model** \| ***χ*²_1_** \| **P-value** \| \| 1 \| MM ⋅ NS \| MM + NS \| 7.652 \| 0.648 \| \| 2 \| MM + NS \| MM \| 95.651 \| 0.104 \| \| 3 \| MM \| 1 \| 226.260 \| **0.013** \| \| Final model: Mean number of eggs per female ~ MM \| \| \| \| \|  \| **Final model coefficients [relative to intercept]** \| ***t*-value** \| **Estimate** \| **Std. Error** \| \| --- \| --- \| --- \| --- \| \| Intercept \| 18.492 \| 3.593 \| 0.194 \| \| Male morph [scrambler] (MM) \| 2.537 \| 0.745 \| 0.294 \| |

| Table S4. Single mating experiment: results of general linear hypothesis testing of contrasts within treatments of the statistically significant two-way interaction between male morph and mating week on the number of laid eggs. *P*-values smaller than 0.05 show that there is a statistically significant difference between the two hyphenated treatment factors within the treatment factor in the first column. Shown estimates (*ê*) and their standard errors (SE) are of the first mentioned treatment factors (in the column header) relative to the second mentioned factor. | |
| --- | --- |
| Interaction: male morph and mating week (P = 0.032) | |
|  | **Male morph: Fighter - Scrambler** |
| Week 1 | *ê* = −1.575, SE = 0.561, *z =* −2.806, *P* = 0.005 |
| Week 2 | *ê* = −0.083, SE = 0.544, *z =* −0.153, *P* = 0.878 |
|  |  |
|  | **Mating week: Week 1 - Week 2** |
| Fighter | *ê* = −0.836, SE = 0.587, *z =* −1.425, *P* = 0.154 |
| Scrambler | *ê* = 0.655, SE = 0.474, *z =* 1.381, *P* = 0.167 |

| Table S5. Single mating experiment: the mean number of eggs laid by females obtained from each of the four stock populations as well as the contrasts between populations obtained by using general linear hypothesis testing. | | | | | | | |
| --- | --- | --- | --- | --- | --- | --- | --- |
|  |  |  |  | ***P*-value difference, with population number** | | | |
|  | **Mean** | **SE** | ***N*** | **I** | **II** | **III** | **IV** |
| Population I | 1.000 | 0.441 | 20 | - | - | - | - |
| Population II | 1.167 | 0.506 | 18 | 1.000 | - | - | - |
| Population III | 1.000 | 0.485 | 17 | 0.624 | 0.587 | - | - |
| Population IV | 0.133 | 0.091 | 15 | 0.077 | 0.084 | 0.302 | - |
